# Effects of Recall and Selection Biases on Modeling Cancer Risk From Mobile Phone Use: Results From a Case–Control Simulation Study

**DOI:** 10.1097/EDE.0000000000001749

**Published:** 2024-05-20

**Authors:** Liacine Bouaoun, Graham Byrnes, Susanna Lagorio, Maria Feychting, Abdellah Abou-Bakre, Rémi Béranger, Joachim Schüz

**Affiliations:** From the a Environment and Lifestyle Epidemiology Branch, International Agency for Research on Cancer, World Health Organization (IARC/WHO), Lyon, France; b Department of Oncology and Molecular Medicine, Istituto Superiore Di Sanità, Rome, Italy; c Unit of Epidemiology, Institute of Environmental Medicine, Karolinska Institutet, Stockholm, Sweden; d Univ Rennes, CHU Rennes, Inserm, EHESP, Irset (Institut de recherche en santé, environnement et travail), UMR_S 1085, Rennes, France.

**Keywords:** Cancer risk, Mobile phone use, Recall errors, Selection bias, Simulations

## Abstract

**Background:**

The largest case–control study (Interphone study) investigating glioma risk related to mobile phone use showed a J-shaped relationship with reduced relative risks for moderate use and a 40% increased relative risk among the 10% heaviest regular mobile phone users, using a categorical risk model based on deciles of lifetime duration of use among ever regular users.

**Methods:**

We conducted Monte Carlo simulations examining whether the reported estimates are compatible with an assumption of no effect of mobile phone use on glioma risk when the various forms of biases present in the Interphone study are accounted for. Four scenarios of sources of error in self-reported mobile phone use were considered, along with selection bias. Input parameters used for simulations were those obtained from Interphone validation studies on reporting accuracy and from using a nonresponse questionnaire.

**Results:**

We found that the scenario simultaneously modeling systematic and random reporting errors produced a J-shaped relationship perfectly compatible with the observed relationship from the main Interphone study with a simulated spurious increased relative risk among heaviest users (odds ratio = 1.91) compared with never regular users. The main determinant for producing this J shape was higher reporting error variance in cases compared with controls, as observed in the validation studies. Selection bias contributed to the reduced risks as well.

**Conclusions:**

Some uncertainty remains, but the evidence from the present simulation study shifts the overall assessment to making it less likely that heavy mobile phone use is causally related to an increased glioma risk.

In 2011, the International Agency for Research on Cancer Monograph Program on the Identification of Carcinogenic Hazards to Humans characterized radiofrequency electromagnetic fields (RF-EMF) as possibly carcinogenic to humans.^1^ The evidence from human studies was classified as limited, due to some positive findings for which bias and error could not be ruled out as a possible explanation.

Until now, the few cohort studies on mobile phone use and the risk of brain tumors have not shown any associations but had applied rather crude exposure assessment and exposure data were not detailed enough to investigate the heaviest mobile phone users separately.^2,3^ Conversely, some of the case–control studies on the same topic have^4,5^ reported positive associations, but simultaneously were more affected by recall and selection biases than the cohorts.

The largest case–control study (Interphone) was carried out according to a common study protocol in 13 countries between 2000 and 2005.^6,7^ Risk analyses in the Interphone study^7^ were performed using one statistical approach only, by defining never regular mobile phone users as the reference category and categorizing the regular mobile phone users (at least one call per week over a period of 6 months or more) into deciles based on the observed distribution of mobile phone use among controls.

While the Interphone study showed an inverse association between glioma risk – the most common type of brain tumor – and regular mobile phone use as compared with never regular use, there was also a positive association with a 40% increased risk among the 10% heaviest users among the regular mobile phone users (about 5% of the total study population of 30- to 59-year olds). In fact, looking at the entire exposure–outcome relationship, a J-shaped risk function was observed, with decreased risks among the lower and moderate mobile phone users and the aforementioned increase in the highest percentile of regular use. Considering the idea that mobile phone use would protect from developing a glioma to be biologically implausible, the Interphone study group interpreted this finding as clear evidence for the presence of bias.^8^

There is now a wide-ranging open debate on whether the positive association seen among the heaviest mobile phone users would also be due to an underlying bias affecting the study. In this regard, it was of particular interest to investigate what types of bias would contribute to creating a spurious J-shaped relationship in the decile-based statistical approach.

Three major biases have been identified in the original Interphone study and its related validation studies.^6–9^ First, selection bias through differential participation among controls was observed using a nonresponse questionnaire,^8^ as there were more mobile phone users among participating than nonparticipating controls, creating a spurious inverse association with overall regular mobile phone use. Second, underestimation of mobile phone use among low users and overestimation by heavy users were observed when comparing self-reported mobile phone use with operator-recorded traffic records, which would lead to an inflation of the association in case of a real exposure–outcome relationship, or if an apparently increased risk is present due to recall and/or other biases.^9^ Third, implausible values of self-reported use as observed in the main Interphone study were more common among glioma cases, which may lead to a spurious positive association,^7^ and refer to a clear example of differential error when the error differs between cases and controls. In this article, we define random measurement error in self-reported mobile phone use as random reporting fluctuations between the observed (self-reported) and the true (traffic records) mobile phone use, since it is in general challenging to precisely recall past mobile phone use. Systematic measurement error in self-reported mobile phone use was due to the observation that there were differences between the observed and true use that differed systematically with the amount of mobile phone use, as introduced above.^9^ We refer to nondifferential measurement error and nondifferential exposure misclassification if the random and systematic measurement error did not differ between cases and controls, and differential if errors did differ by disease status.

Previous simulations assessing the effects of errors in self-reported mobile phone use on risk estimations have shown that random errors have a larger impact on the risk estimates than systematic errors and differential errors had limited additional impact in the presence of large random errors.^10^ Selection bias resulting from underrepresentation of unexposed controls led to a J-shaped exposure–outcome relationship, with risk apparently decreasing at low-to-moderate exposure levels.^7,8^ Nevertheless, a limitation of using the previous simulation results in the evaluation of bias is the more general assessment of the potential impact of measurement error and bias not closely matching the statistical approach of the main risk analysis, mainly because those simulations were made before the risk analysis was reported.

In this work, the main objective was to investigate whether the observed exposure–outcome relationship across the deciles of cumulative mobile phone use and the risk of glioma was compatible with a modeled exposure–outcome relationship applying different recall error types as observed in the validation studies in the absence of any real effect. Therefore, we were only using the identical approach as applied to and reported from the Interphone main risk analysis,^7^ namely categorical analysis of deciles of lifetime duration of use in regular mobile phone users compared with never regular users as reference group, i.e., the main risk analysis as included in the respective health risk assessments and evidence syntheses such as the International Agency for Research on Cancer Monographs.^1^ Specifically, we performed Monte Carlo simulations aimed at examining if the increased risk of glioma observed among the 10% heaviest mobile phone users, as reported in the Interphone study,^7^ could have been observed assuming no real effect of mobile use. These simulations were expected to provide further insight into the interplay of the previously discussed three major biases.

## MATERIALS AND METHODS

### Study Population

The Interphone case–control study was conducted by 16 centers in 13 countries between 2000 and 2005. Detailed information on study methods has been reported elsewhere.^6,7^ Two Interphone exposure validation studies were conducted in parallel to the main Interphone study in some of the participating countries, enabling us to quantify the effects of different types of measurement errors in the assessment of mobile phone use.^6^ The first Interphone validation study enrolled a total of 690 healthy volunteers from 12 countries^11^ as an independent study sample from the main Interphone study, while the second validation study collected retrospectively additional data from 508 participants (296 controls and 212 cases) from three countries of the main Interphone case–control study for whom the respective data could be obtained.^9^ From these two validation studies, we obtained the true (hereafter referred to as *X*) and observed (*Y*) mobile phone exposure derived from operator-recorded traffic records and from self-reported mobile phone use data, respectively, for two available mobile phone use exposure metrics: the monthly cumulative number of calls and the monthly cumulative duration of calls. Validation of the representativeness of participants was part of the main Interphone case–control study, as a short nonresponse questionnaire was provided to subjects who refused to participate in the main study, applied in most of the Interphone study centers.^8^ Among nonparticipants of the main study, 21% responded to the nonresponse questionnaire (26% among controls and 9% among cases). Quantitative measures of mobile phone use as in the main study were not collected in the nonresponse questionnaire, but instead more generic mobile phone use data were available, for example, the year a participant first started using a mobile phone.

### Statistical Analysis

We log-transformed all mobile phone use metrics (both self-reported and operator-recorded number and duration of calls) prior to the statistical analyses, as the log-transformed values nearly followed a normal distribution. For each metric, we also calculated the log ratio *T* (or log recall error) defined by the log ratio of the self-reported to the operator-recorded mobile phone data.

We conducted Monte Carlo simulation analyses to examine the effects of multiple types of mobile phone use recall errors on glioma risk estimates in a case–control study design, using categorized mobile phone use exposure variables. Simulations were set up to mimic the full model used in the original Interphone study and are briefly described below. Further details of the simulations are also provided in the eAppendix 2; http://links.lww.com/EDE/C144. We conducted analyses using the R statistical software, version 3.6.1.

#### Simulation Study: Generating Model

We generated 5000 main datasets (*D*, *X*, *Y*) of 3000 subjects (2000 controls and 1000 cases) with a true (*X*) and an observed (error-prone; *Y*) mobile phone use exposure, and an outcome (case–control) status *D*. We drew a random case–control dataset (*D*, *X*, *Y*) of 3000 subjects as follows:

1. A regular mobile phone user status was randomly generated from a Bernoulli random variable, Bin(P), with probability P based on the distribution of exposure among controls as observed in the main Interphone study.^7^
2. Among regular mobile phone users,
  - The true exposure *X* was randomly generated from a normal distribution: $N\left(\mu_{X},\sigma_{X}^{2}\right)$, where $\mu_{X}$ and $\sigma_{X}^{2}$ are the mean and the variance of the true exposure levels, respectively, and are obtained as described below.
  - The observed exposure *Y* prone to error was generated, conditional on the true exposure *X*, from a normal distribution, $N\left(X+\tau +\gamma X_{c},\sigma_{T}^{2}\right)$, where τ denotes the average value of the error T (between the observed *Y* and the true exposure *X*), γ is the slope reflecting the strength between the error-prone and the true exposure *X* (with the mean of *X*;$X_{c}$), which enables us to test the deviation from the average value, and $\sigma_{T}^{2}$ the random variance;
3. We finally generated the outcome status *D* from a Bernoulli random variable conditional on *X*, so that $P\left(D=1|X\right)=expit\left(\alpha^{*}+\beta^{*}\times X\right)$, where $\alpha^{*}$ relates to the (baseline) probability of glioma in the absence of exposure (i.e., among nonregular users) while $OR^{*}=exp\left(\beta^{*}\right)$ is the true odds ratio (OR).

The definition of “regular mobile phone use” followed that of Interphone,^7^ that is, at least one call per week for a period of at least 6 months. We assumed nonregular mobile phone users to have provided accurate data, which is of “zero” exposure, assuming no exposure measurement error.

We generated simulated case–control datasets under both the null hypothesis (*H*_0_) of no real effect ($OR^{*}=1)$ and also – for comparison purposes – under the alternative hypothesis (*H*_1_) assuming a real effect. Under *H*_1_, an $OR^{*}$ of 1.30 was arbitrarily chosen as a possible glioma risk estimate associated with mobile phone use if there was one. ORs were expressed per “delta” increase in exposure, where “delta” corresponded to the difference of exposure between the last decile (heavy users) and the first decile (light users) of the exposure level (see below). We also investigated the situation assuming that the error was associated with the true level of use (γ ≠ 0), as it was reported previously that low users underestimated their use while heavy users overestimated it.^9^

#### Fitting Model

First, we categorized the continuous exposures *X* and *Y* into deciles based on their distribution among regular mobile phone users for each exposure metric separately, exactly as done in the Interphone main analysis,^7^ as explained earlier. Then, for each simulated dataset, we fitted separate logistic regression models of *D* on the categorical true exposure *X* (hereafter referred to as the “true” estimator), and of *D* on the error-prone categorical exposure *Y* (“naive”), in order to estimate the true parameter $\beta^{*}$, with nonregular users of mobile phone as the reference category. We repeated these steps 5000 times, one for each mobile phone use metric.

#### Evaluation

For each decile and each type of estimator, we summarized the simulation results by computing several statistical measures including the mean of the coefficient estimates, along with its 95% confidence interval and the coverage probability. Since the log-transformed cumulative number and duration of calls were similar and because the increased risk seen in the main study was only with duration of calls, we only present findings from the analyses based on duration of calls as the main mobile phone exposure metric. We observed similar results based on the number of calls (eTables 9 and 10 and eFigures 2 and 3; http://links.lww.com/EDE/C143 in eAppendix 1; http://links.lww.com/EDE/C143).

#### Scenarios Investigated as Sources of Error in Mobile Phone Use

We investigated four main plausible scenarios of (measurement) errors in self-reported mobile phone use, as truly observed in the Interphone validation studies (eTables 1 and 5; http://links.lww.com/EDE/C143), all based on the abovementioned main equation: $Y∼N\left(X+\tau +\gamma X_{c},\sigma_{T}^{2}\right)$. In scenario 1, we assessed the combined effects of greater random and systematic errors in cases than in controls. This is the scenario closest to the reality evidenced by the Interphone validation studies.^9,11^ In scenario 2 (only random error but still differential), we assumed cases to have greater random error with a larger standard deviation error than controls but with the same mean, reflecting the possibility that the accuracy of reporting mobile phone use may be affected by their health status. In scenario 3, we investigated the effect of greater systematic error in cases than in controls with a fixed random error. The scenario 4 assumed only random error not differing between cases and controls (nondifferential).

#### Input Simulation Parameters

We used the posterior predictive distribution parameters of a Bayesian hierarchical linear model as input parameters of mobile phone use in simulations that accounted for mobile phone use heterogeneity found across the Interphone study countries (eTable 8; http://links.lww.com/EDE/C143 and eAppendix3; http://links.lww.com/EDE/C145). These parameters reflected credible distributions representing the most realistic and wide variety of situations observed in the Interphone validation studies.

#### Selection Bias Through Time Since First Regular Use

Participation rates in the Interphone study were rather low and varied strongly between cases and controls, and across study centers.^8^ The overall response rates were 71% among glioma cases, but only 53% among controls. Since the amount of mobile phone use was not available from the nonresponse questionnaire (as otherwise participation in the nonresponse questionnaire would have been lower), the year, since participants started using a mobile phone regularly, was used in our simulations. Hence, risk analyses were extended by using the year of the start of regularly using a mobile phone divided into four categories: ≤1992, 1993–1997, 1998–2000, ≥2001. Nonregular users served as the reference category. We compared the dates of first use between interviewees and responders from the nonresponse questionnaire and also reported the ratios *W* of proportions between the two groups in each exposure category, which allowed the use of the bias-correction formula of Greenland^12^ to obtain bias-corrected risk estimates (eAppendix2; http://links.lww.com/EDE/C144).

## RESULTS

### Descriptive Data From the Interphone Exposure Validation Studies

Based on both Interphone validation studies, the estimated mean and standard deviation of mobile phone use data, as well as the ratio *T*, are shown in eTable 2; http://links.lww.com/EDE/C143. On average, both cases and controls underestimated their number of calls by up to 0.21 (on the log scale) and overestimated call duration by up to 0.34 (on the log scale), consistent with already published findings.^9,11^ Similar findings were also supported using untransformed data (eTable 6; http://links.lww.com/EDE/C143). eTable 3; http://links.lww.com/EDE/C143 presents estimates by participating countries. There was heterogeneity (linear mixed models, likelihood ratio test (LRT) *P* values <0.05) in errors (*T*) across countries, especially among countries from the first validation study. Interestingly, in the Italian validation case–control study, on average, cases overestimated their number of calls by 0.04 while controls underestimated them by 0.12 (eTable 7; http://links.lww.com/EDE/C143), which was different from the two other countries (Australia, Canada).

### Monte Carlo Simulation Results of Modeling Errors in Mobile Phone Use Recall

In the following section, we focus on results under the null assumption of no real effect, unless otherwise stated. We start with scenario 4, which is the one based on the true observations from the validation studies, followed by the others for a better understanding of the impact of the individual different measurement errors.

#### Scenario 1: Differential Random and Systematic Errors – Scenario Based on Real Validation Studies

This is the most interesting scenario as reflecting closely the observations from the validation studies,^9,11^ combining greater random and systematic errors among cases than controls, where the main equation reduces to


$$\begin{array}{l} Y∼\left\{ \begin{array}{l} N\left(X,\sigma_{T_{0}}^{2}\right)\;amongcontrols\\ N\left(X+\tau_{1}+\gamma_{1}X_{c,1},1.1\times \sigma_{T_{0}}^{2}\right)amongcases \end{array}\right.. \end{array}$$

We observed a J-shaped curve between deciles of exposure (relative to nonregular mobile phone users) and (log) risk estimates with a greater underestimation of risk among the lower users followed by a slight underestimation among the moderate users (medium deciles) and then ending with a clear overestimation of risks for the heavier users (the last deciles), creating a spurious positive association among the 10% heaviest users (Table 1 and Figure D). The last decile (d10) related to heavy mobile phone users exhibited a particularly poor performance with a strong overestimation of risk (OR = 1.91 [95% confidence interval = 1.22, 3.00]) where we estimated the type-1 error rate as 93% and the coverage probability as 7%. The greater the random error in cases compared to controls, the more pronounced became the J-shape curve (eFigure 5; http://links.lww.com/EDE/C143).

**Table 1.** Simulation Results of Measurement Errors in Mobile Phone Use on Risk Estimates for Each Decile Under the Null Hypothesis (*H*_0_) of No Effect; by Scenario Along With the True Estimator; Duration of Calls

Number of Calls		Deciles of Exposure									
Scenario^a^	Scenario Number	D1	D2	D3	D4	D5	D6	D7	D8	D9	D10
Without error (true estimator)											
Coverage	-	95.3	95.5	94.1	96.2	94.4	95.0	94.4	95.1	94.8	95.2
Type-1 error		4.7	4.5	5.9	3.8	5.6	5.0	5.6	4.9	5.2	4.9
Differential systematic and random^b^	1										
Coverage		63.8	66.5	70.6	79.3	86.9	92.1	93.9	88.5	61.8	6.6
Type-1 error		36.2	33.5	29.4	20.7	13.1	7.9	6.1	11.5	38.2	93.4
Differential random^c^	2										
Coverage		71.8	94.8	94.3	92.7	91.2	91.2	92.8	94.6	94.7	71.4
Type-1 error		28.2	5.1	5.7	7.3	8.8	8.8	7.2	5.4	5.3	28.6
Differential systematic^d^	3										
Coverage		31.0	59.9	78.1	89.0	92.0	94.9	90.1	80.5	61.8	25.7
Type-1 error		69.0	40.1	21.9	11.0	8.0	5.1	9.9	19.5	38.2	74.3
Random error^e^	4										
Coverage		89.3	95.4	95.1	93.4	93.6	93.9	94.3	94.6	94.5	88.9
Type-1 error		10.7	4.6	4.9	6.6	6.4	6.1	5.7	5.4	5.5	11.1

a In all scenarios, nonregular mobile phone users served as the reference category. The true OR (OR*) used for generating the model is supposed to be equal to 1.0.

b Differential random and systematic scenario: cases have greater random (10% more) and average systematic (*τ* = 0.34) error than controls, and the error increases with the level of use (*γ* = 0.02). Random standard deviation error is set to 1.22 among controls ($\sigma_{T_{0}}$).

c Differential random scenario; cases have greater random error than controls (average standard deviations ratio between cases and controls equal to 1.1).

d Differential systematic scenario: cases have greater average systematic error than controls (expectation *τ* = 0.34) and the error increased with the level of use (*γ* = 0.02). Random error is kept at a constant level (of 1.28) and similar among cases and controls.

e Random error scenario: the random standard deviation $\sigma_{T}$ is set to 1.22.

**Figure.**
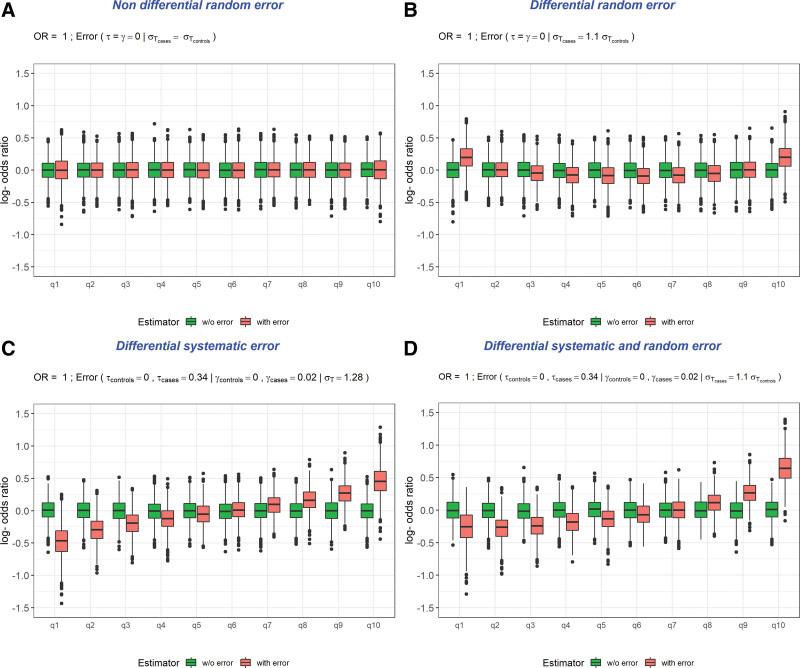
Boxplots (over 5000 replicates) of (log) risk estimates associated with deciles of exposure (total duration of calls) in the absence of an effect (*H*_0_; OR* = 1.0) for different scenarios.^a^ True (without error) and naive (with error) estimators.^a^In all scenarios, nonregular mobile phone users served as the reference category. The true OR (OR*) used for generating the model is supposed to be equal to 1.0. Scenarios were: differential random and systematic scenario: cases have greater random (10% more) and average systematic (*τ* = 0.34) error than controls, with the error increases with the level of use (*γ* = 0.02) and the random standard deviation error is set to 1.22 among controls ($\sigma_{T_{0}}$) (D; scenario 1); differential random scenario; cases have greater random error than controls (average standard deviations ratio between cases and controls equal to 1.1) (B; scenario 2); differential systematic scenario: cases have greater average systematic error than controls (expectation *τ* = 0.34) with the error increased with the level of use (*γ* = 0.02) and the random error is kept at a constant level (of 1.28) and similar among cases and controls (C; scenario 3); random error scenario: the random standard deviation $\sigma_{T}$ was set to 1.22 (A; scenario 4).

Under *H*_1_, with $OR^{*}=1.3$, among the heavier users the power was close to 100% while the coverage dropped to 18% with a clear overestimation of risk (observed OR = 2.89 vs. expected OR = 1.70) (eTable 4 and eFigure 1D; http://links.lww.com/EDE/C143). We noticed a poor coverage among light (d1) mobile phone users. We observed a similar J-shaped curve, also reinforcing the active effect of greater differential random errors among cases and the limited impact of the magnitude of the true effect size on the shape of the curve (eFigures 1 and 5; http://links.lww.com/EDE/C143).

#### Scenario 2: Only Differential Random Errors

Where cases have greater random error than controls $\left(\sigma_{T_{1}}=1.1\sigma_{T_{0}}\right)$, with the expected error value (τ) set to 0 and with no deviation between the error and true value (γ=0), the main equation reduces to:


$$\begin{array}{l} Y∼\left\{ \begin{array}{l} N\left(X,\sigma_{T_{0}}^{2}\right)\;amongcontrols\\ N\left(X,1.1\times \sigma_{T_{0}}^{2}\right)\;amongcases \end{array}\right.. \end{array}$$

We observed a U-shaped response but still with a high right-hand tail (Table 1 and Figure B). The type-1 error rate increased to around 28% while the coverage probability decreased to 71% for left-hand and right-hand tail deciles corresponding to light and heavy users. Again here, the greater the random error in cases compared to controls, the more pronounced the U-shape curve (eFigure 4; http://links.lww.com/EDE/C143). This pattern was somewhat similar to that observed in the mixed-errors scenario (scenario 4) with the presence of greater differential random errors among cases (eFigure 5; http://links.lww.com/EDE/C143).

#### Scenario 3: Differential Systematic Errors

Here cases have greater systematic error than controls with errors increasing with the level of use while the random error is kept at the same level between cases and controls, hence the main equation reduces to:

$$Y∼\left\{ \begin{array}{l} N\left(X,\sigma_{T}^{2}\right)\;amongcontrols\\ N\left(X+\tau_{1}+\gamma_{1}X_{c,1},\sigma_{T}^{2}\right)\;amongcases \end{array}\right.$$

Overestimation of exposure among cases – with both average systematic error and a positive association between the error and the level of use – resulted in an underestimation of risks in the first deciles and an overestimation in the last deciles with an apparent symmetry of risk estimates around the medium decile (Table 1 and Figure C). We observed a clear difference in risks among light users compared with the previous scenario 2, suggesting random error has a higher impact than systematic error in explaining the mixed-errors scenario. We observed a strong degradation of both type-1 error rates and coverage probabilities, especially among heavy users where the average risk estimate was away from the null, the type-1 error rate was 74%, and the coverage probability collapsed to 26%. When the error was unrelated to the true level of mobile phone use, similar patterns were observed in the risk estimates suggesting again the limited impact of the level of association introduced between the error and the true level of use.

#### Scenario 4: Only Nondifferential Errors

The presence of only nondifferential random error, where the main equation reduces to $Y∼N\left(X,\sigma_{T}^{2}\right),$ had a limited impact on risk estimates with a flat exposure–outcome curve where the type-1 error rates and the coverage probabilities were close to their nominal values (5% and 95% for the type-1 error rate and coverage probability, respectively) (Table 1 and Figure A). These measures were slightly degraded for light and heavy regular mobile phone users. This scenario suggests that nondifferential errors had no major impact.

Under *H*_1_, there was an inflation of the risk estimates away from the null for the deciles below the median and attenuation for the deciles higher than the median (eFigure 1A; http://links.lww.com/EDE/C143) with a slight underestimation of the risk estimate among the heaviest users (observed OR = 1.51 vs. expected OR = 1.70). Coverage probabilities showed good performance (>80%) and the power linearly increased with increasing decile of exposure (from about 54% to 67%) (eTable 4; http://links.lww.com/EDE/C143). The magnitude of the random error introduced had a limited impact on the results.

#### Selection Bias by Time Since First Regular Use

The ratios *W* of proportions (of exposure) between interviewed participants and responders from the nonresponse questionnaire for each exposure category are presented in Table 2 for cases and controls separately and selection probabilities are shown in Table 3. For both cases and controls, regular mobile phone users were overrepresented (*W* > 1) among interviewed participants, especially those who started using a mobile phone in 1993–1997, with the exception of subjects in the more recent category of exposure start year (from 2001+). Never regular mobile phone users were less likely than users to agree to participate in the Interphone study, notably among controls. We also observed major differences in selection probabilities between cases and controls, whereas cases were likely to participate with a probability close to one. The analyses assuming values of underparticipation of nonregular mobile phone users among controls and among those with more recent start dates (as observed in the Interphone study) showed narrower and less attenuated estimates for those who started recently (from 2001+) than for those who started using a mobile phone earlier. The introduction of selection bias also clearly underestimated risk estimates.

**Table 2.** Ratios of Percentage of Exposure (Year of Start Using a Mobile Phone) Among Interphone Interviewees to Responders From the Nonresponse Questionnaire, Stratified by Case–Control Status; Interphone Study

Exposure	Levels	*W*^a^ Control	*W*^a^ Case	*W* Case–Control
Nonregular users	-	0.70	0.71	1.01
Regular users	≥2001	0.84	0.90	1.07
	1998–2000	1.39	1.05	0.76
	1993–1997	1.91	1.97	1.03
	≤1992	1.72	1.32	0.77

Study centers with data on start year using a mobile phone regularly: Australia, Canada-Montreal, Canada-Vancouver, Finland, Germany, Israel, Italy, Norway, and Sweden for controls and Australia, Finland, Germany, Israel, Italy, and Sweden for cases.

a Ratio of percentage of exposure among interviewed to responders from the nonresponse questionnaire from Interphone study.

**Table 3.** Participation Probabilities Into the Interphone Study by Exposure (Year of Start Using a Mobile Phone Regularly) and by Case–Control Status

Exposure	Levels	Control	Case
Nonregular users	-	0.67	0.93
Regular users	≥2001	0.70	0.94
	1998–2000	0.80	0.95
	1993–1997	0.84	0.97
	≤1992	0.83	0.96

Study centers with data on start year using a mobile phone regularly: Australia, Canada-Montreal, Canada-Vancouver, Finland, Germany, Israel, Italy, Norway, and Sweden for controls and Australia, Finland, Germany, Israel, Italy, and Sweden for cases. Participation rates were 75.8% among controls and 95% among cases.

## DISCUSSION

In this study, we conducted Monte Carlo simulation analyses to examine whether the effects of applying various types of uncertainties in self-reported mobile phone use on the estimation of glioma risk in the absence of any real effect could be compatible with the observed J-shaped exposure–outcome function in the main Interphone case–control study^7^ (eFigure 6; http://links.lww.com/EDE/C143). To our knowledge, this is the first simulation study quantifying the risk of glioma with categorical mobile phone use exposure metrics when various types of bias and uncertainties present in the multinational Interphone study are accounted for. Using the exact same categorical exposure measures here as were used in the risk analysis of the Interphone study enabled us to go further than several previous simulations that made more general assessments of possible biases and observed that random measurement errors attenuate the risk estimates toward the null.^9–11,13^ Based on the findings from two Interphone validation studies on the quality of self-reported mobile phone use, we found that our simulation model (scenario 4 from above) applying both the observed greater random and the observed greater systematic measurement errors in cases than in controls in the absence of any real effect was perfectly compatible with the observed J-shaped exposure–outcome relationship and the increased risk among the 10% heaviest regular mobile phone users, as seen in the main Interphone study^7^ (eFigure 6; http://links.lww.com/EDE/C143). Consequently, this strengthens the hypothesis that the risk increase observed among heavy regular mobile phone users in the main Interphone study is an artifact and most likely due to random and systematic variations of errors in self-reported mobile phone use in the absence of any real effect.

Our simulations showed that among the biases present in the main Interphone study many affect the risk estimation, even though quantitatively two major ones could explain the observed J-shape in its entirety, even if there was no true association between mobile phone use and glioma risk. First, the selection bias leading to overrepresentation of regular mobile phone users in controls aligns well with the observed inverse association among light or moderate mobile phone users (as to both the direction and the magnitude of impact). Second, the systematic exposure measurement error in self-reported mobile phone use (consisting of underreporting of actual use by low users and overestimation by heavy users), with its larger variance in cases than controls (differential error), would strongly contribute to the creation of a J-shaped exposure–outcome relationship. This would be in a way that the right tail of the curve would not only approach the null value but instead create a spurious positive association only in the very highest decile of exposure. This effect was even enlarged because more cases than controls reported implausibly high amounts of mobile phone use as well. Especially the finding of the presence of a larger variance among cases than controls in the systematic error in self-reports to our knowledge has not been described before and therefore also never been applied before in any error simulations.^8–11^

The present simulation study also has several limitations. First, it is the possible presence of unaccounted residual uncertainties, since the bias assessment is based on the samples used in the validation studies, and not on the entire study population of Interphone,^8–11^ and the validation studies themselves may therefore not be free of bias. Hence, a true effect of the exposure cannot totally be refuted – as also seen in our simulations with the presence of a real effect (eFigures 1 and 5; http://links.lww.com/EDE/C143) – even though, as said, the most likely bias scenario matches very well the observed results of the main study. In the original papers from the Interphone study,^7^ the interpretation of bias was rather dismissed, mainly because the mean difference between operator and self-reported data was not significantly different between cases and controls. However, as explained above, which was the main new finding from this simulation study, it was the variance ratio that mattered that was never compared before, neither individually nor between cases and controls. The slope of the error between operator-recorded and self-reported data versus the average was also not adequately tested.

Second, the scale of mobile phone use data utilized in the present study was not fully comparable to that of the main Interphone study, since only monthly cumulative mobile phone use data of both the number and duration of calls were available from the two Interphone validation studies while lifetime cumulative exposures were reported from the main Interphone case–control study. So, this did not allow us to make exactly equal comparisons regarding the magnitude of risk estimates but as analogous patterns of results were seen it is unlikely they would be different for lifetime exposure.

Third, we were also not able to account for the amount of mobile phone use in simulation analyses of selection bias and therefore also not able to examine the combined effect of selection bias and measurement errors since the amount of use was not available from the nonresponse questionnaire. Time since first mobile phone use was the only exposure detail that was assessed in the nonresponse questionnaire. Although selection bias and measurement errors are likely to operate together, it was not possible to quantitatively explore the way they interact in the decile-based risk analyses.

Last, we assumed that responders from the nonresponse questionnaire were representative of all nonparticipants, including those who were not asked or refused to complete the nonresponse questionnaire, although we could not rule out that these “other” nonparticipants may have different mobile phone use profiles compared to their nonresponse questionnaire counterparts. The simulation study was also limited by its lack of controlling for other sources of bias like potential confounders so that residual confounding may still be possible. However, confounding bias was thoroughly investigated in the main Interphone study and no credible candidate was identified.^7^

Our simulations showed that the aforementioned issues are critical. In fact, the J-shaped exposure–outcome curve results from both an increase in variance of self-reported use in the cases versus the controls and from a different slope in their nonlinear relation, that is, two of the issues not analyzed in the previously published Interphone validation study papers.^8–11^ The increased variance places more cases than controls into the extreme use categories (at either end of the range), while the difference in slope modifies this slightly so that the exaggeration is greatest in the highest (or lowest) mobile phone user categories. Moving more cases into these categories implies that the estimated effect will be greatest for cases, with a reduction in risk suggested in the intermediate categories. In the Bayesian analyses, it was found that the increase in variance was common across all countries. In the three countries providing data for the recall bias validation study,^9^ this resulted in an amplified “U”- or “J”-shaped response similar to that which is overall proposed. The difference in the operator-recorded to self-reported use between cases and controls was small compared with that between the three countries, while the role of the increased variance to the increase apparent exposure rates in the extreme categories was dominant. It is unlikely that results from further case–control studies will resolve the question of whether mobile phone use is related to an increased glioma risk due to limitations inherent in this particular study design; additional prospective cohort studies like the COSMOS study^14,15^ are designed to overcome many of these limitations. While some error in reporting will inevitably remain as it appears challenging to remember accurately the exact amount of mobile phone use, the fact that this information is collected before developing a glioma and only heavy users versus light users within the cohort are compared eliminates two of the major error sources of the main Interphone case–control study.

Vast majority of mobile phone users at the time Interphone was conducted were users of the second generation of mobile phone technology (Global System for Mobile Communications [GSM]) while today the fifth generation (5G) has already been launched, which has a large impact on the RF-EMF exposures from mobile phone use. Whereas, in general, numbers of calls have increased and age at first use is lower, leading to some increase in cumulative exposure, the output power of mobile phones has gone dramatically down and more common use of video calls and headsets leads to less calls with the phone directly hold to the head, leading to a substantial decrease in cumulative exposure, so that, on average, RF-EMF exposure to the head is lower than in the past. On the other hand, past exposures are important determinants in the development of chronic diseases such as cancer, so the public health relevance of the Interphone findings and their interpretation remains. Due to the changing nature of exposure, monitoring of time trends of glioma incidence rates is important, with the most recent one not showing any change in time trends compatible with the hypothesis of an increased risk from mobile phone use.^16^

## CONCLUSION

In this simulation study, the most likely bias scenario, evidenced by data from validation studies on the quality of self-reported mobile phone use and produced under the assumption of no association between mobile phone use and glioma risk, showed a J-shaped relationship similar to that observed in the main Interphone study; with a reduced risk among light and moderate mobile phone users along with an increased risk in heavy mobile phone users. This strengthens bias as an explanation for the observation in the main study and weakens the evidence that the increased risk observed in the heaviest mobile phone users is causal. Ultimately, however, this research question will only be resolved with new data such as data coming from prospective cohort studies minimizing these types of bias.

## ACKNOWLEDGMENTS

The authors are grateful to Mrs. Monika Moissonnier for her skillful data management and the participants of the INTERPHONE study and its validation studies who gave so generously of their time.

This work would not have been possible without the original Interphone study. A summary of this project (coordinator: Dr. Elisabeth Cardis) and contact details of the collaborators are found at https://interphone.iarc.fr/list-of-collaborators/.

## Supplementary Material


