# Supplementary material for: Effects of Recall and Selection Biases on Modeling Cancer Risk From Mobile Phone Use: Results From a Case–Control Simulation Study
Source: Epidemiology. 2024 May 20;35(4):437–46. doi: 10.1097/EDE.0000000000001749 (PMC11191551; doi:10.1097/EDE.0000000000001749)
Supplement: Supplementary file 1 [file ede-35-437-s001.docx]

# Supplementary Methods

### Data generating model

We generated random case-control datasets (D, X, Y) of 3000 subjects where the issue of extreme exposure values - which were not structurally excluded by the choice of a semi-unbounded distribution - was addressed by removing these subjects based on a global three-step approach. First, a large dataset of 200,000 subjects was initially created when generating a simulated case-control dataset (D, X, Y), aiming to maximize the likelihood of subsequently selecting the aforementioned subset of 3000 subjects. Second, we left out subjects with true exposure X greater than the maximum exposure level in the operator-recorded dataset (the maxima recorded were, on the log-scale, 7.8 and 8.5 for the number and duration of calls, respectively). Third, we finally drew a random case-control dataset (D, X, Y) of 3000 subjects from this large dataset as described in the main document.

### Evaluation: Statistical measures computed

For each decile and each type of estimator, the simulation results were summarized by computing the mean of the coefficient estimates, along with its 95% confidence interval (CI), along with the coverage probability statistics over all simulated data sets. The 95% CIs were calculated by taking the 2.5^th^ and the 97.5^th^ percentiles of the distribution of the parameter estimates. The coverage probability corresponds to the proportion of estimated confidence intervals that contained the expected true value. The expected risk estimates were determined for all decile categories, and calculated by multiplying the mean of the true exposure within each decile by the true odds ratio (${OR}^{*}$) applied. The log-risk estimates were then graphically described using boxplots across all simulated datasets, separately for each decile of mobile phone use and each of the two estimators, and overlapped with the expected true estimates to enable comparisons, where appropriate. Ultimately, we tested the two-tailed hypothesis where the null hypothesis (H_0_) was that the coefficient estimate (of the log-OR) is zero, using a significance level of 5%. Calculating the probability of rejecting H_0_ enabled us to assess either the false-positive (type-1) error rate (in the absence of a real effect with ${OR}^{*}=1$), or the study power (in the presence of a real effect).

### Scenarios investigated

Four scenarios of (measurement) errors in mobile phone use recall were investigated - as observed in the Interphone validation studies - that were based on a combination of random, systematic, differential, and non-differential errors (eTables 1 and 5). In scenario 1, we assumed that cases were prone to both greater random and systematic recall errors than controls where not only random errors are present among cases and controls but also systematic differences in the expectation of exposure. This is the scenario closest to reality, as this is what was observed in the Interphone validation studies ^9,10^, enabling a quantification of the potential biases. In scenario 2, cases were assumed to have only greater random recall errors than controls but with the same mean. Indeed, cases from the Interphone study were interviewed under quite different conditions than controls (cases mainly in hospitals while controls mainly at home), likely involving both recall bias and potential differential errors due to the biological effects of the glioma. In scenario 3, we investigated the effect of differential systematic exposure measurement errors with a fixed random error, assuming greater recall errors in cases than in controls. This implemented systematic exposure measurement error model (scenarios 3) does not correspond to a purely systematic exposure measurement error with a random component $\sigma_{T}^{2}$ set to 0, but rather to a real situation where there is always some noise in the exposure with $\sigma_{T}^{2}>0$ fixed. In scenario 4 (also known as the "classical measurement error model") we assumed random recall errors usually with zero mean and constant variance. We investigated this well-known situation briefly as a validation of our simulations since this is well understood in the literature on regression calibration ^13^, resulting in an attenuation of the estimate towards the null effect (in the presence of a real effect).

### Input simulation parameters and Bayesian analysis of mobile phone use across multi-countries

A Bayesian analysis was carried out to reconstruct a credible range of effect sizes given the results observed in the main Interphone study, while allowing for the biases presented in this work. We considered a Bayesian hierarchical analysis of the mobile phone use data variation (heterogeneity) across study countries. The primary data relating case and control errors in self-reporting of past mobile phone use was available only from three countries of one of the two Interphone validation studies: Australia, Canada and Italy. Therefore, estimates of differences of these data between cases and controls can come only from these countries. The hierarchical (or multilevel) normal model assumes, for the log-transformed exposure data *y_j_* of country *j* and data category *k*:


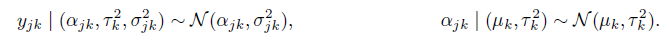


where σ_jk_ correspond to standard deviations and α_jk_ are country-specific effects. The heterogeneity parameter τ*_k_* measures the between-country variance for each exposure category, so τ*_k_* = 0 implies the model reduces to a fixed-effect meta-analysis model.

Since our primary interest was to estimate total excess variance, and not to estimate country-specific variance, we integrated over the α_jk_ for fixed *k* to obtain general estimates for the category means *μ_k_* and their variances σ^2^_jk_ :


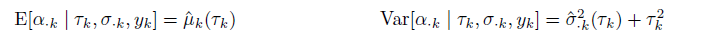


where the estimators, *μ_k_*, are conditional posterior effects as functions of the heterogeneity parameters τ*_k_*. We then used the posterior predictive distribution parameters, α_._*_k_*, of the Bayes estimates as input parameters in risk modelling to generate (log-) risk estimates, using both means and standard deviations. We applied this strategy separately for the cumulative number of calls and the cumulative duration of calls.

In addition, α^*^ was set to log(0.006), so that the risk to develop glioma of each simulated subject was assumed to be equal to 0.6% in the absence of mobile phone use exposure, as observed in the main Interphone study ^7^. In a similar way, the mobile phone user status probability P was set to P = 0.64 based on the distribution of exposure among controls ^7^. Subjects with unlikely operator-recorded exposures were excluded, as mentioned earlier. We also investigated both situations assuming that the error was uncorrelated ($\gamma$ = 0), and correlated ($\gamma$ ≠ 0), with the true level of use.

## Selection bias through time since first regular use

Whenever possible, subjects refusing to participate to the main Interphone study were asked to complete a short non-response questionnaire, aimed at evaluating whether they differed from those who agreed to take part in the main Interphone study. Again, the completion of the non-response questionnaire data differed across study centres and between cases and controls. Overall, 8.7% of cases and 25.8% of controls responded to the non-response questionnaire. We then considered the year of start using a mobile phone regularly - from both responders from the non-response questionnaire and interviewees of the main Interphone study - into the selection process in order to investigate the effects of selection bias in the association between mobile phone use and cancer risk. We assumed that responders from the non-response questionnaire were representative of all non-participants, including those who were not asked or refused to complete the non-response questionnaire.

Let *K*, *D* and *S* be the exposure in categories, the outcome variable (case/control) and the selection (participation) of the Interphone study indicator, respectively. *S* = 1 denotes interviewed participants and *S* = 0 denotes responders from the non-response questionnaire. The categorical exposure *K* indicating the year of start using a mobile phone regularly was divided into 5 distinct categories: non-users, and among users; ≤1992, 1993-1997, 1998-200, ≥ 2001.

In this case, we obtained bias-corrected cancer risk estimates using the following formula, from Greenland ^14^ :


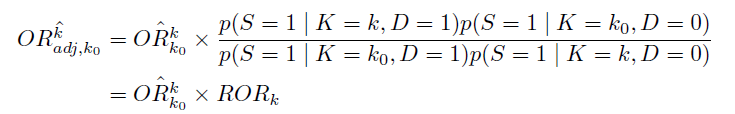


where OR^k^_k0_ is the risk estimate comparing the level k to the reference level k_0_ (=non-regular users), p(S = 1 | K =k, D = j) the selection probability and ROR_k_ the so-called selection bias factor.

ROR := (ROR_k1_ , . . . , ROR_k4_) is the corresponding vector for each level of K.
