# Supplementary material for: Effects of Recall and Selection Biases on Modeling Cancer Risk From Mobile Phone Use: Results From a Case–Control Simulation Study
Source: Epidemiology. 2024 May 20;35(4):437–46. doi: 10.1097/EDE.0000000000001749 (PMC11191551; doi:10.1097/EDE.0000000000001749)

**eTable 1**: Scenarios investigated as sources of measurement error in mobile phone use ^a^.

| Scenario | Description | Key parameters |
| --- | --- | --- |
| 1 | **Differential systematic and differential random error** with Y ~ $N$(X + $\tau_{status}$+ ${\gamma_{status}X}_{c, \mathrm{status}}$, $\sigma_{T_{status}}^{2}$), where $\tau_{0}=\gamma_{0}=0$for controls, $\tau_{1}$and $\gamma_{1}$varying for cases varying for cases, and $\sigma_{T_{1}}$= k*$\sigma_{T_{0}}$, k$\in\left\{ 1, 1.1, 1.2, 1.3, 1.6 \right\}$ and $\sigma_{T_{0}}$fixed | $\tau_{1}, \gamma_{1}$ and $\sigma_{T_{1}}$ |
| 2 | **Differential random error with greater random error among cases than controls** with Y ~ $N$(X, $\sigma_{T_{status}}^{2}$) where $\sigma_{T_{1}}^{2}$= k*$\sigma_{T_{0}}$, k$\in\left\{ 1, 1.1, 1.2, 1.3, 1.6 \right\}$ and $\sigma_{T_{0}}$fixed | $\sigma_{T_{1}}$ |
| 3 | **Differential systematic over estimation of mobile phone use among cases** and fixed random error with Y ~ $N$(X + $\tau_{status}$+ ${\gamma_{status}X}_{c, status}$, $\sigma_{T}^{2}$) where $\tau_{0}=\gamma_{0}=0$for controls, $\tau_{1}$and $\gamma_{1}$varying for cases, while $\sigma_{T}$is fixed | $\tau_{1}$and $\gamma_{1}$ |
| 4 | **Non-differential random error** with Y ~$N$(X, $\sigma_{T}^{2}$) where $\sigma_{T}$ varying | $\sigma_{T}$ |

^a^ In scenarios 1-3 where differential errors between cases and controls are introduced, two sets of parameters were defined: one for the controls (indexed by the lowercase 0) and one for the cases (by the lowercase 1). All scenarios are based on the main generic equation: Y ~ $N$(X + τ + γ$X_{c}$, $\sigma_{T}^{2}$) where τ denotes the average value of the error T (between the observed Y and the true exposure X), γ is the slope reflecting the strength between the error-prone and the true exposure X (with the mean of X; $X_{c}$), and $\sigma_{T}^{2}$ is the variance of the T.

**eTable 2**: Means and standard deviations (SD) of mobile phone use data: self-reported, operator-recorded and log-ratio of self-reported to operator-recorded data; by study and exposure metric (number and duration of calls). Interphone validation studies.

|  |  | Number of calls | | | Durations of calls | | |
| --- | --- | --- | --- | --- | --- | --- | --- |
|  | N | Mean | SD | Ratio; mean (SD) | Mean | SD | Ratio; mean (SD) |
| Study 1^a^ |  | | | | | | |
| Operator-recorded | 690 | 4.25 | 1.13 | -0.09 (1.04) | 4.80 | 1.36 | 0.38 (1.30) |
| Self-reported | 690 | 4.16 | 1.36 |  | 5.17 | 1.75 |  |
| Study 2^b^ |  | | | | | | |
| **By status** |  | | | | | | |
| Controls: Operator-recorded | 296 | 4.46 | 0.96 | -0.21 (0.97) | 4.86 | 1.12 | 0.33 (1.23) |
| Controls: Self-reported | 296 | 4.25 | 1.25 |  | 5.19 | 1.57 |  |
| Cases: Operator-recorded | 212 | 4.44 | 0.94 | -0.21 (1.03) | 4.90 | 1.09 | 0.34 (1.30) |
| Cases: Self-reported | 212 | 4.23 | 1.36 |  | 5.24 | 1.71 |  |
| **Overall** |  | | | | | | |
| Operator-recorded | 508 | 4.45 | 0.95 | -0.21 (0.99) | 4.87 | 1.11 | 0.33 (1.26) |
| Self-reported | 508 | 4.24 | 1.30 |  | 5.21 | 1.63 |  |

^a^ Vrijheid M et al. (2006).

^b^ Vrijheid M et al. (2009).

**eTable 3**: Log-ratios of self-reported to operator-recorded by study, country and exposure metric (number and duration of calls). Interphone validation studies.

|  |  | **Number of calls** | | **Durations of calls** | |
| --- | --- | --- | --- | --- | --- |
|  |  | Ratio | | Ratio | |
|  | N | Mean | SD | Mean | SD |
| Study 1^a^ |  | | | | |
| **By country** |  |  |  |  |  |
| Australia | 44 | 0.06 | 0.82 | 0.33 | 0.97 |
| Denmark | 46 | -0.22 | 0.62 | 0.29 | 0.97 |
| Finland | 78 | 0.07 | 0.78 | 0.48 | 1.22 |
| France | 70 | -0.10 | 0.95 | 0.47 | 1.29 |
| Germany | 75 | -0.24 | 1.15 | 0.03 | 1.48 |
| Israel | 41 | -0.10 | 1.01 | 0.36 | 1.35 |
| Italy | 89 | 0.10 | 1.01 | 0.78 | 1.17 |
| New Zealand | 20 | -0.87 | 0.94 | -0.35 | 0.88 |
| Norway | 46 | -0.79 | 0.88 | -0.58 | 1.16 |
| Sweden | 46 | -0.16 | 0.95 | 0.44 | 1.23 |
| UK North OP* | 91 | 0.47 | 1.30 | 0.96 | 1.47 |
| UK North SMP* | 44 | -0.49 | 0.84 | 0.06 | 0.95 |
| **Overall** | 690 | -0.09 | 1.04 | 0.38 | 1.30 |
| Study 2^b^ |  | | | | |
| **By country** |  |  |  |  |  |
| Australia | 192 | -0.44 | 0.97 | 0.26 | 1.18 |
| Canada | 131 | -0.08 | 0.95 | 0.46 | 1.2 |
| Italy | 185 | -0.05 | 1.00 | 0.32 | 1.36 |
| **Overall** | 508 | -0.21 | 0.99 | 0.33 | 1.26 |

^*^ In the UK-North study, two separate studies were carried out: one using only software modified phones (SMP) and one using only mobile phone operators’ records (UK North OP).

^a^ Vrijheid M et al. (2006).

^b^ Vrijheid M et al. (2009).

**eTable 4**: Simulation results of measurement errors in mobile phone use on risk estimates for each decile under the alternative hypothesis (H1; ${OR}^{*}=1.3$) with the presence of a real effect; by scenario along with the true estimator. Duration of calls.

| Number of calls |  | Deciles of exposure | | | | | | | | | |
| --- | --- | --- | --- | --- | --- | --- | --- | --- | --- | --- | --- |
| Scenario ^a^ | Scenario number | D1 | D2 | D3 | D4 | D5 | D6 | D7 | D8 | D9 | D10 |
| **Without error (true estimator)** |  |  |  |  |  |  |  |  |  |  |  |
| Coverage | - | 95.0 | 94.7 | 95.5 | 95.3 | 95.0 | 94.8 | 95.6 | 94.9 | 95.3 | 94.2 |
| Power |  | 30.3 | 41.9 | 47.2 | 55.2 | 61.7 | 67.2 | 71.6 | 77.2 | 80.5 | 90.2 |
| **Differential systematic and random** ^b^ | 1 |  |  |  |  |  |  |  |  |  |  |
| Coverage |  | 78.3 | 77.8 | 78.8 | 83.6 | 86.2 | 91.7 | 93.6 | 90.9 | 73.4 | 17.5 |
| Power |  | 18.9 | 10.2 | 14.9 | 24.1 | 32.6 | 49.1 | 67.6 | 87.2 | 97.6 | 99.9 |
| **Differential random** ^c^ | 2 |  |  |  |  |  |  |  |  |  |  |
| Coverage |  | 50.5 | 92.8 | 95.5 | 92.7 | 92.2 | 90.6 | 90.6 | 91.0 | 93.7 | 86.1 |
| Power |  | 86.0 | 60.0 | 46.1 | 44.9 | 43.8 | 43.0 | 46.9 | 54.2 | 70.0 | 93.2 |
| **Differential systematic** ^d^ | 3 |  |  |  |  |  |  |  |  |  |  |
| Coverage |  | 47.3 | 72.9 | 83.5 | 89.9 | 95.0 | 94.1 | 91.8 | 87.5 | 72.4 | 42.3 |
| Power |  | 18.1 | 9.0 | 17.5 | 35.5 | 50.0 | 67.1 | 82.6 | 91.7 | 97.3 | 99.7 |
| **Random error** ^e^ | 4 |  |  |  |  |  |  |  |  |  |  |
| Coverage |  | 82.0 | 92.8 | 92.9 | 93.4 | 94.0 | 93.6 | 94.4 | 94.7 | 92.8 | 82.0 |
| Power |  | 53.5 | 58.6 | 60.2 | 59.8 | 61.4 | 64.5 | 66.2 | 66.1 | 69.9 | 67.0 |

^a^ In all scenarios, non-regular mobile phone users served as the reference category. The true OR (${OR}^{*}$) used for generating the model is

supposed to be equal to 1.3.

^b^ Differential random and systematic scenario: cases have greater random (10% more) and average systematic (τ = 0.34) error than controls, and the error increases with the level of use (γ = 0.02). Random standard deviation error is set to 1.22 among controls ($\sigma_{T_{0}}$).

^c^ Differential random scenario; cases have greater random error than controls (average standard deviations ratio between cases and controls equal to 1.1).

^d^ Differential systematic scenario: cases have greater average systematic error than controls (expectation τ = 0.34) and the error increased with the level of use (γ = 0.02). Random error is kept at a constant level (of 1.28) and similar among cases and controls.

^e^ Random error scenario: the random standard deviation $\sigma_{T}$ is set to 1.22.

**eTable 5**: Scenarios of measurement errors in self-reported mobile phone use based on random/systematic and non-differential/differential errors.

| *Scenarios* | **Non-differential** | **Differential** |
| --- | --- | --- |
| **Random and Systematic** | *~~-~~* | Scenario 1 |
| **Random** | Scenario 4 | Scenario 2 |
| **Systematic** | *~~-~~* | Scenario 3 |

**eTable 6**: Median^c^ and standard deviations (SD) of mobile phone use data: self-reported, operator-recorded and ratio of self-reported to operator-recorded data. Untransformed (original) data; by study and exposure metric (number and duration (in minutes) of calls). Interphone validation studies.

|  |  | Number of calls | | | Durations of calls | | | | |  |
| --- | --- | --- | --- | --- | --- | --- | --- | --- | --- | --- |
|  | N | Median | SD | Ratio; median (SD) | Median | | SD | | Ratio; median (SD) | |
| Study 1^a^ |  | | | | | | | | |  |
| Operator-recorded | 690 | 76.6 | 139.4 | 0.90 (8.84) | 137.9 | | 333.6 | | 1.52 (29.1) | |
| Self-reported | 690 | 76.0 | 262 |  | 201.3 | | 3983 | |  |  |
| Study 2^b^ |  | | | | | | | | |  |
| **By status** |  | | | | | | | | |  |
| Controls: Operator-recorded | 296 | 83.4 | 161.5 | 0.82 (1.52) | 130.5 | 296.8 | | 1.36 (5.38) | |  |
| Controls: Self-reported | 296 | 82.2 | 172 |  | 183.1 | 1260 | |  |  |  |
| Cases: Operator-recorded | 212 | 83.8 | 139.2 | 0.87 (2.10) | 138.9 | 323.7 | | 1.37 (7.19) | |  |
| Cases: Self-reported | 212 | 75.8 | 298 |  | 176.7 | 1710 | |  |  |  |
| **Overall** |  | | | | | | | | |  |
| Operator-recorded | 508 | 83.4 | 152.5 | 0.83 (1.79) | 131.5 | 308 | | 1.36 (6.20) | |  |
| Self-reported | 508 | 75.8 | 233.0 |  | 180.8 | 1466 | |  |  |  |

^a^ Vrijheid M et al. (2006).

^b^ Vrijheid M et al. (2009).

^c^ Since mobile phone use data have large right-skewed distributions, medians instead of means are reported.

**eTable 7**: Means of mobile phone use measurement error (log ratio of self-reported to operator-recorded data); by country, case/control status and exposure metric (number and duration of calls). Interphone case-control validation study.

|  |  | Cases | Controls |
| --- | --- | --- | --- |
|  | N | **Number of calls** | |
| Study 2^b^ |  |  |  |
| **By country** |  |  |  |
| Australia | 192 | -0.52 | -0.39 |
| Canada | 131 | -0.11 | -0.05 |
| Italy | 185 | 0.04 | -0.12 |
| **Overall** | 508 | -0.21 | -0.21 |
|  |  | **Durations of calls** | |
| Study 2^b^ |  |  |  |
| **By country** |  |  |  |
| Australia | 192 | 0.21 | 0.29 |
| Canada | 131 | 0.26 | 0.60 |
| Italy | 185 | 0.51 | 0.17 |
| **Overall** | 508 | 0.34 | 0.33 |

^b^ Vrijheid M et al. (2009).

**eTable 8**: Means (τ), slope ($\gamma$) and standard deviations ($\sigma)$ of measurement error in mobile phone use (log ratio of self-reported to operator-recorded data); by study, case/control status and exposure metric (number and duration of calls). Interphone validation studies.

|  |  | Mean / expectation (τ) | Slope ($\gamma)$ | Standard deviation $\sigma$ | Difference in expectation (τ) between cases and controls | Difference in slope ($\gamma)$ between cases and controls | Standard deviation $(\sigma)$ ratio between cases and controls |
| --- | --- | --- | --- | --- | --- | --- | --- |
|  | **N** | **Number of calls** | | | | | |
| Study 1^a^ |  |  |  |  |  |  |  |
| **Overall** | 690 | -0.09 | -0.20 | 1.01 | - | - | - |
| Study 2^b^ |  |  |  |  |  |  |  |
| **By status** |  |  |  |  |  |  |  |
| Controls | 296 | -0.21 | -0.16 | 0.96 | -0.0010 | 0.12 | 1.08 |
| Cases | 212 | -0.21 | -0.04 | 1.03 |  |  |  |
| **Overall** | 508 | -0.21 | -0.11 | 0.99 | - | - | - |
|  |  | **Durations of calls** | | | | | |
| Study 1^a^ |  |  |  |  |  |  |  |
| **Overall** | 690 | 0.38 | -0.13 | 1.29 | - | - | - |
| Study 2^b^ |  |  |  |  |  |  |  |
| **By status** |  |  |  |  |  |  |  |
| Controls | 296 | 0.33 | -0.12 | 1.22 | 0.01 | 0.14 | 1.07 |
| Cases | 212 | 0.34 | 0.02 | 1.30 |  |  |  |
| **Overall** | 508 | 0.33 | -0.06 | 1.25 | - | - | - |

^b^ Vrijheid M et al. (2009).

**eTable 9**: Simulation results of measurement errors in mobile phone use on risk estimates for each decile under the null hypothesis (H0) of no effect; by scenario along with the true estimator. Number of calls.

| Number of calls |  | Deciles of exposure | | | | | | | | | |
| --- | --- | --- | --- | --- | --- | --- | --- | --- | --- | --- | --- |
| Scenario ^a^ | Scenario number | D1 | D2 | D3 | D4 | D5 | D6 | D7 | D8 | D9 | D10 |
| **Without error (true estimator)** |  |  |  |  |  |  |  |  |  |  |  |
| Coverage | - | 95.3 | 95.0 | 95.3 | 95.7 | 95.0 | 94.5 | 95.5 | 95.5 | 95.1 | 95.6 |
| Type-1 error |  | 4.7 | 5.0 | 4.7 | 4.3 | 5.1 | 5.5 | 4.5 | 4.5 | 4.9 | 4.4 |
| **Differential systematic and random** ^b^ | 1 |  |  |  |  |  |  |  |  |  |  |
| Coverage |  | 30.8 | 27.0 | 37.8 | 52.6 | 68.7 | 88.7 | 94.5 | 72.6 | 23.5 | 2.4 |
| Type-1 error |  | 69.2 | 73.0 | 62.2 | 47.4 | 31.3 | 11.3 | 5.5 | 27.4 | 76.5 | 97.6 |
| **Differential random** ^c^ | 2 |  |  |  |  |  |  |  |  |  |  |
| Coverage |  | 73.1 | 94.2 | 93.8 | 91.7 | 91.6 | 92.2 | 93.2 | 95.2 | 95.3 | 73.8 |
| Type-1 error |  | 26.9 | 5.8 | 6.2 | 8.3 | 8.3 | 7.8 | 6.9 | 4.9 | 4.7 | 26.2 |
| **Differential systematic** ^d^ | 3 |  |  |  |  |  |  |  |  |  |  |
| Coverage |  | 15.5 | 26.5 | 42.3 | 59.3 | 79.9 | 92.5 | 91.4 | 62.8 | 24.7 | 6.4 |
| Type-1 error |  | 84.5 | 73.5 | 57.7 | 40.7 | 20.1 | 7.5 | 8.6 | 37.2 | 75.3 | 93.6 |
| **Random error** ^e^ | 4 |  |  |  |  |  |  |  |  |  |  |
| Coverage |  | 89.6 | 95.6 | 94.0 | 93.3 | 93.9 | 93.4 | 94.0 | 94.2 | 94.9 | 89.3 |
| Type-1 error |  | 10.3 | 4.4 | 6.0 | 6.7 | 6.1 | 6.6 | 6.0 | 5.8 | 5.1 | 10.7 |

^a^ In all scenarios, non-regular mobile phone users served as the reference category. The true OR (${OR}^{*}$) used for generating the model is supposed to be equal to 1.0.

^b^ Differential random and systematic scenario: cases have greater random (10% more) and average systematic (τ = 0.21) error than controls, and the error increases with the level of use (γ = 0.54). Random standard deviation error is set to 0.96 among controls ($\sigma_{T_{0}}$).

^c^ Differential random scenario: cases have greater random error than controls (average standard deviations ratio between cases and controls equal to 1.1).

^d^ Differential systematic scenario: cases have greater average systematic error than controls (expectation τ = 0.21) and the error increased with the level of use (γ = 0.54). Random error is kept at a constant level (of 1.01) and similar among cases and controls.

^e^ Random error scenario: the random standard deviation $\sigma_{T}$ is set to 0.97.

**eTable 10**: Simulation results of measurement errors in mobile phone use on risk estimates for each decile under the alternative hypothesis (H1; ${OR}^{*}=1.3$) with the presence of a real effect; by scenario along with the true estimator. Number of calls.

| Number of calls |  | Deciles of exposure | | | | | | | | | |
| --- | --- | --- | --- | --- | --- | --- | --- | --- | --- | --- | --- |
| Scenario ^a^ | Scenario number | D1 | D2 | D3 | D4 | D5 | D6 | D7 | D8 | D9 | D10 |
| **Without error (true estimator)** |  |  |  |  |  |  |  |  |  |  |  |
| Coverage | - | 93.9 | 93.9 | 95.3 | 95.0 | 94.3 | 94.4 | 95.2 | 95.2 | 94.5 | 95.0 |
| Power |  | 38.1 | 55.4 | 62.5 | 67.2 | 72.9 | 79.1 | 82.7 | 85.8 | 90.1 | 96.0 |
| **Differential systematic and random** ^b^ | 1 |  |  |  |  |  |  |  |  |  |  |
| Coverage |  | 57.2 | 50.6 | 53.1 | 62.8 | 72.1 | 86.4 | 92.4 | 86.4 | 43.0 | 4.5 |
| Power |  | 26.3 | 15.3 | 15.2 | 14.7 | 27.7 | 49.2 | 75.7 | 95.4 | 99.1 | 99.8 |
| **Differential random** ^c^ | 2 |  |  |  |  |  |  |  |  |  |  |
| Coverage |  | 52.1 | 92.9 | 94.8 | 92.7 | 91.8 | 90.9 | 89.8 | 91.6 | 94.0 | 85.5 |
| Power |  | 90.8 | 72.4 | 61.4 | 56.7 | 56.2 | 58.9 | 61.1 | 69.8 | 84.4 | 97.4 |
| **Differential systematic** ^d^ | 3 |  |  |  |  |  |  |  |  |  |  |
| Coverage |  | 36.3 | 44.7 | 56.7 | 69.3 | 80.7 | 91.3 | 93.0 | 80.4 | 43.1 | 10.9 |
| Power |  | 31.6 | 17.7 | 17.1 | 23.3 | 39.9 | 65.9 | 87.5 | 96.8 | 99.2 | 99.3 |
| **Random error** ^e^ | 1 |  |  |  |  |  |  |  |  |  |  |
| Coverage |  | 88.7 | 93.2 | 94.2 | 94.3 | 94.8 | 94.4 | 95.0 | 95.0 | 94.1 | 90.5 |
| Power |  | 65.1 | 70.3 | 72.1 | 74.6 | 77.3 | 75.8 | 78.5 | 79.7 | 82.8 | 85.5 |

^a^ In all scenarios, non-regular mobile phone users served as the reference category. The true OR (${OR}^{*}$) used for generating the model is

supposed to be equal to 1.3.

^b^ Differential random and systematic scenario: cases have greater random (10% more) and average systematic (τ = 0.21) error than controls, and the error increases with the level of use (γ = 0.54). Random standard deviation error is set to 0.96 among controls ($\sigma_{T_{0}}$).

^c^ Differential random scenario; cases have greater random error than controls (average standard deviations ratio between cases and controls equal to 1.1).

^d^ Differential systematic scenario: cases have greater average systematic error than controls (expectation τ = 0.21) and the error increased with the level of use (γ = 0.54). Random error is kept at a constant level (of 1.01) and similar among cases and controls.

^e^ Random error scenario: the random standard deviation $\sigma_{T}$ is set to 0.97.

**eFigure 1**: Boxplots (over 5000 replicates) of (log-) risk estimates associated with deciles of exposure under the presence of a real effect (H1; ${OR}^{*}=1.3$) for different scenarios^a^. Duration of calls. True (green) and naïve (pink) estimators.


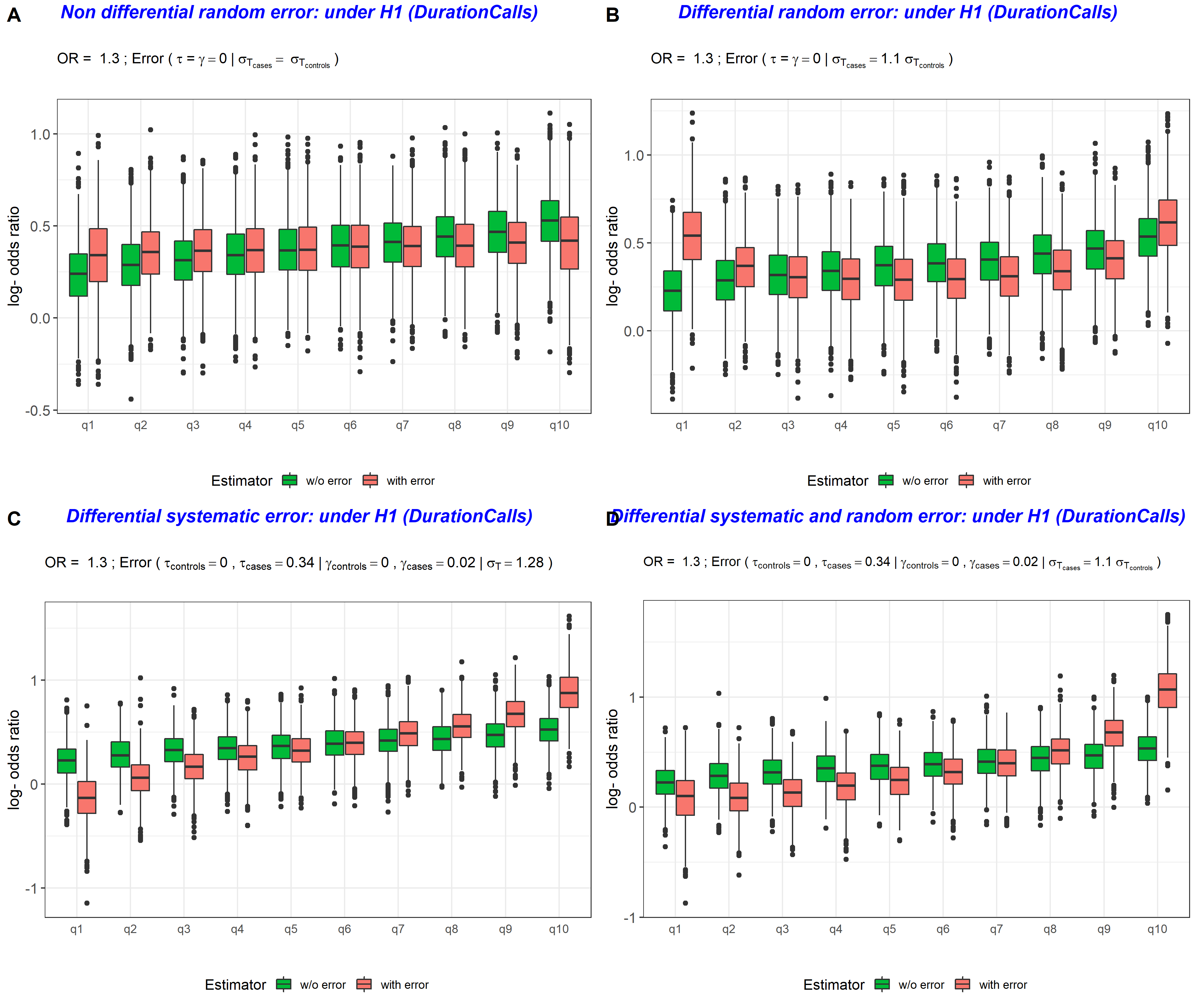
 ^a^ In all scenarios, non-regular mobile phone users served as the reference category. The true OR (${OR}^{*}$) used for generating the model is supposed to be equal to 1.3.

Scenarios were; differential random and systematic scenario: cases have greater random (10% more) and average systematic (τ = 0.34) error than controls, with the error increases with the level of use (γ = 0.02) and the random standard deviation error is set to 1.22 among controls ($\sigma_{T_{0}}$) **(D; Scenario 1)**; differential random scenario; cases have greater random error than controls (average standard deviations ratio between cases and controls equal to 1.1) **(B; Scenario 2)**; differential systematic scenario: cases have greater average systematic error than controls (expectation τ = 0.34) with the error increased with the level of use (γ = 0.02) and the random error is kept at a constant level (of 1.28) and similar among cases and controls **(C; Scenario 3)**; random error scenario: the random standard deviation $\sigma_{T}$ was set to 1.22 (**A; Scenario 4**).

**eFigure 2**: Boxplots (over 5000 replicates) of (log-) risk estimates associated with deciles of exposure (total number of calls) in the absence of an effect (H_0_; ${OR}^{*}=1.0$) for different scenarios^a^. True (green) and naïve (pink) estimators.


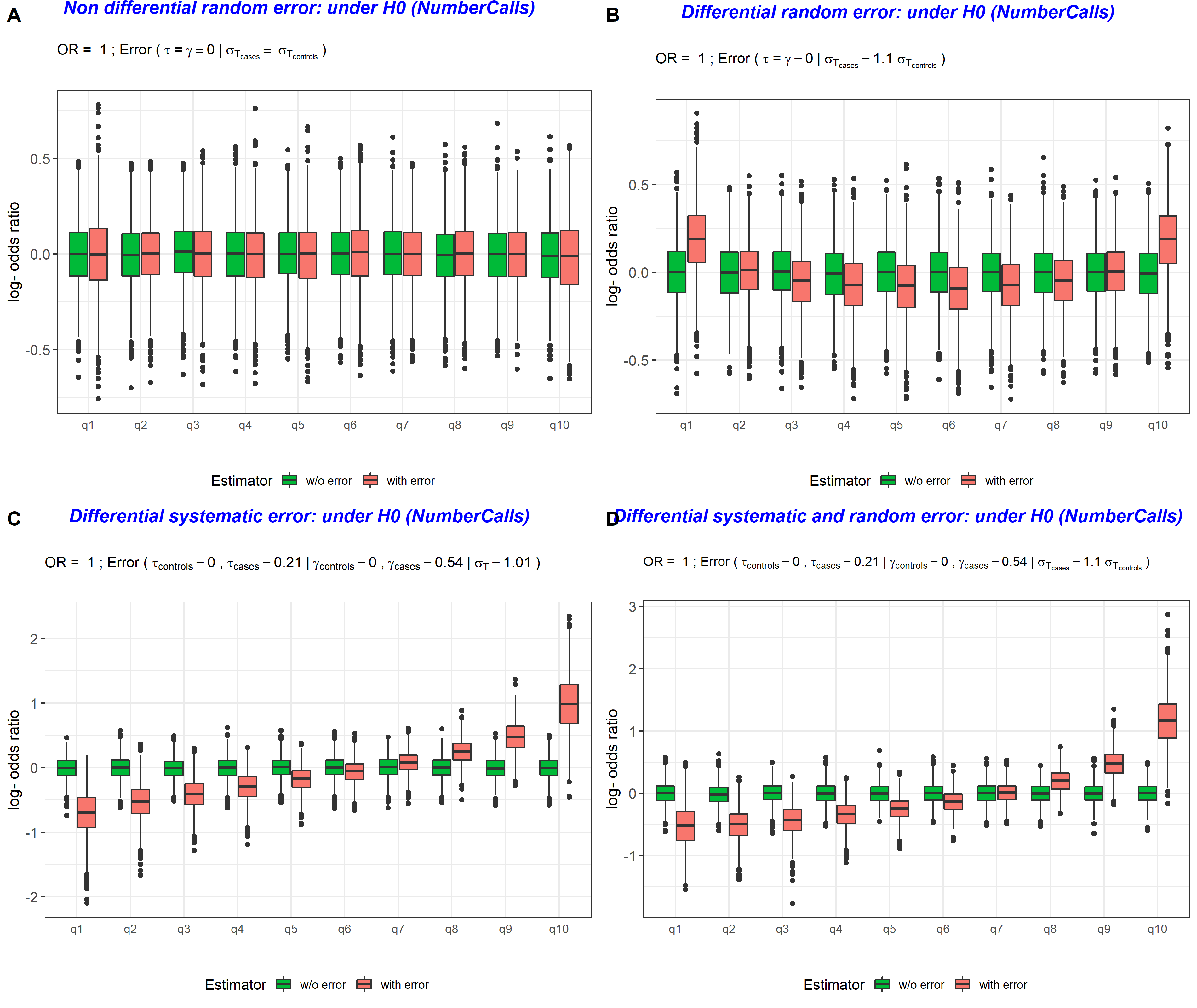
^a^ In all scenarios, non-regular mobile phone users served as the reference category. The true OR (${OR}^{*}$) used for generating the model is supposed to be equal to 1.0.

Scenarios were; differential random and systematic scenario: cases have greater random (10% more) and average systematic (τ = 0.21) error than controls, with the error increases with the level of use (γ = 0.54) and the random standard deviation error is set to 0.96 among controls ($\sigma_{T_{0}}$) **(D; Scenario 1)**; differential random scenario; cases have greater random error than controls (average standard deviations ratio between cases and controls equal to 1.1) **(B; Scenario 2)**; differential systematic scenario: cases have greater average systematic error than controls (expectation τ = 0.21) with the error increased with the level of use (γ = 0.54) and the random error is kept at a constant level (of 1.01) and similar among cases and controls **(C; Scenario 3)**; random error scenario: the random standard deviation $\sigma_{T}$ was set to 0.97 **(A; Scenario 4)**.

**eFigure 3**: Boxplots (over 5000 replicates) of (log-) risk estimates associated with deciles of exposure under the presence of a real effect (H1; ${OR}^{*}=1.3$) for different scenarios^a^. Total number of calls. True (green) and naïve (pink) estimators.


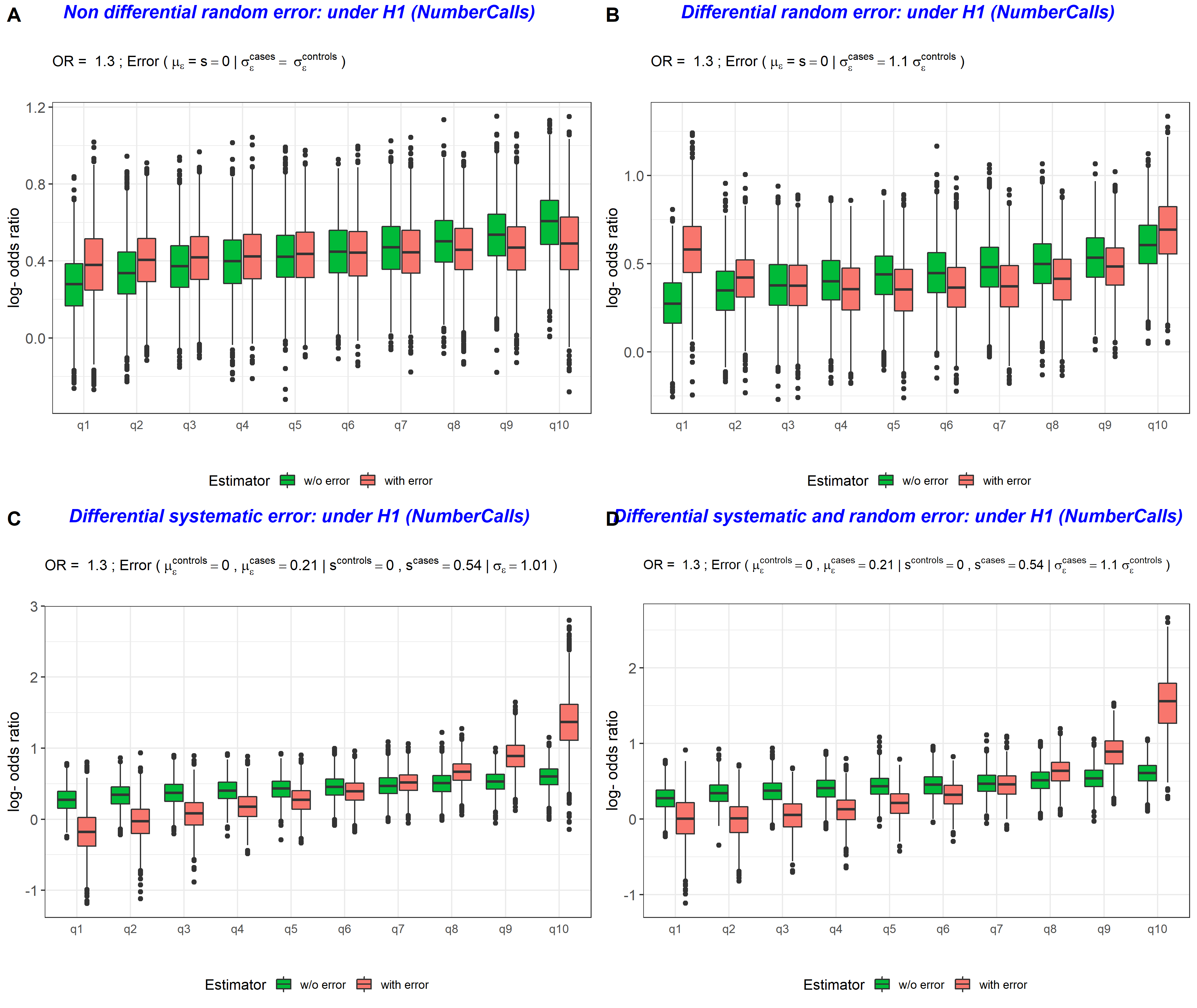
 ^a^ In all scenarios, non-regular mobile phone users served as the reference category. The true OR (${OR}^{*}$) used for generating the model is supposed to be equal to 1.3.

Scenarios were; differential random and systematic scenario: cases have greater random (10% more) and average systematic (τ = 0.21) error than controls, with the error increases with the level of use (γ = 0.54) and the random standard deviation error is set to 0.96 among controls ($\sigma_{T_{0}}$) **(D; Scenario 1)**; differential random scenario; cases have greater random error than controls (average standard deviations ratio between cases and controls equal to 1.1) **(B; Scenario 2)**; differential systematic scenario: cases have greater average systematic error than controls (expectation τ = 0.21) with the error increased with the level of use (γ = 0.54) and the random error is kept at a constant level (of 1.01) and similar among cases and controls **(C; Scenario 3)**; random error scenario: the random standard deviation $\sigma_{T}$ was set to 0.97 **(A; Scenario 4)**.

**eFigure 4**: Boxplots (over 5000 replicates) of (log-) risk estimates associated with deciles of exposure (duration of calls) in the absence of an effect (H_0_; ${OR}^{*}=1.0$)for differential random measurement error scenario^a^ by average standard deviations ratio between cases and controls $\left( k \in\left\{ 1.1, 1.2, 1.3, 1.6 \right\} \right)$. True (green) and naïve (pink) estimators.


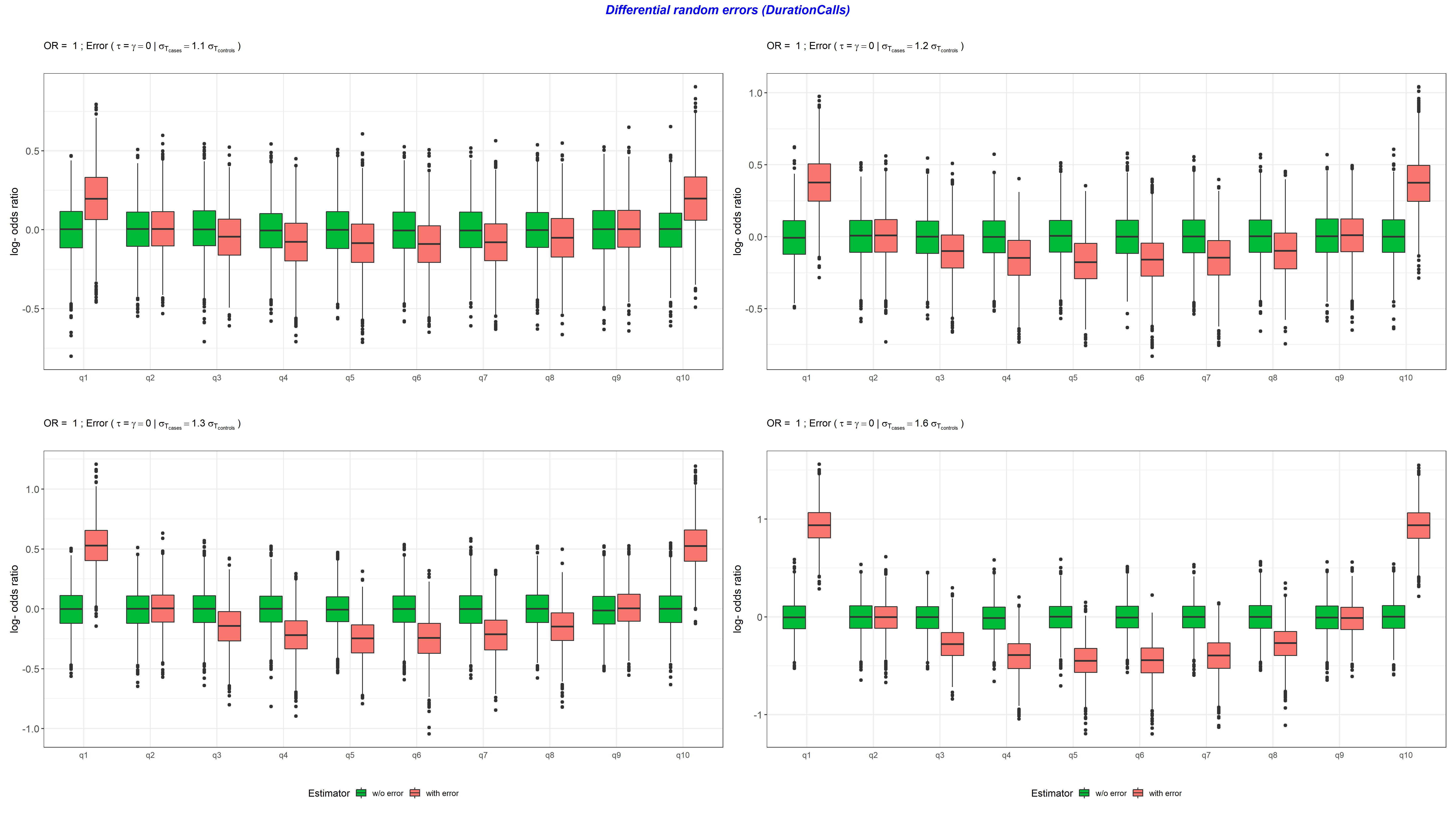
^a^ Non-regular mobile phone users served as the reference category. The true OR (${OR}^{*}$) used for generating the model is supposed to be equal to 1.0. Cases have greater random error than controls (from 10% (upper left window) to 60% (bottom right) more) where the random standard deviation error is set to 1.22 among controls ($\sigma_{T_{0}}$) **(Scenario 2)**.

**eFigure 5**: Boxplots (over 5000 replicates) of (log-) risk estimates associated with deciles of exposure by true ${OR}^{*}$ (rows) and average standard deviations ratios between cases and controls $k$ (columns) for both differential systematic and random measurement error scenario^a^. Total duration of calls. True (green) and naïve (pink) estimators.

^
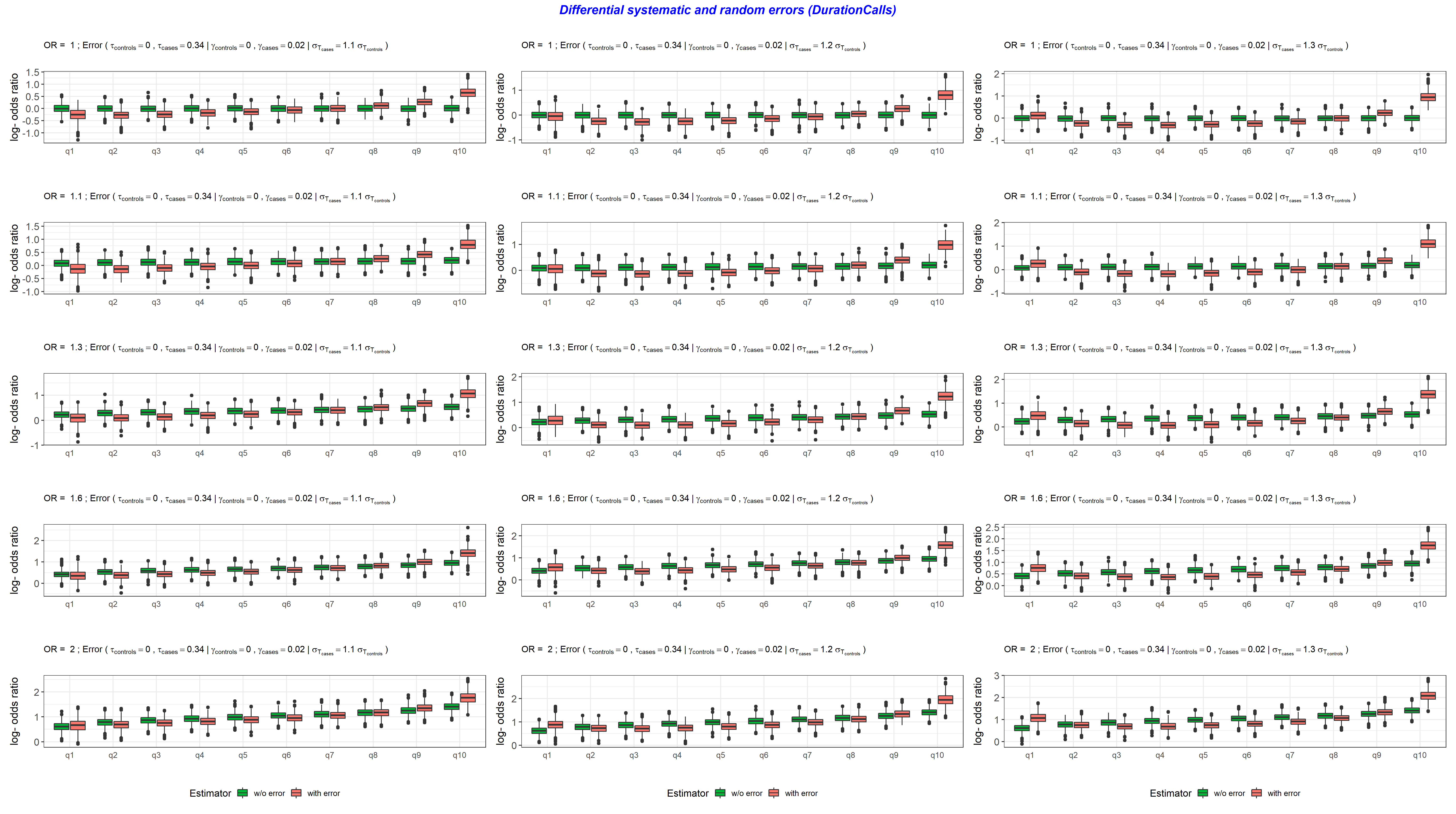
^

^a^ Non-regular mobile phone users served as the reference category. The true OR (${OR}^{*}$), in rows, varies from 1.1 to 2.0. Cases have greater random (columns, from 10% to 30% more) and average systematic (τ = 0.21) error than controls, with the error increases with the level of use (γ = 0.54) and the random standard deviation error is set to 0.96 among controls ($\sigma_{T_{0}}$) **(Scenario 1)**.

**eFigure 6**: Log- odds ratios of the risk of glioma according to the lifetime cumulative hours of mobile phone use (categorized exposure in deciles, log-scale) in the main Interphone multinational case-control study. Non-regular mobile phone users are the reference category.


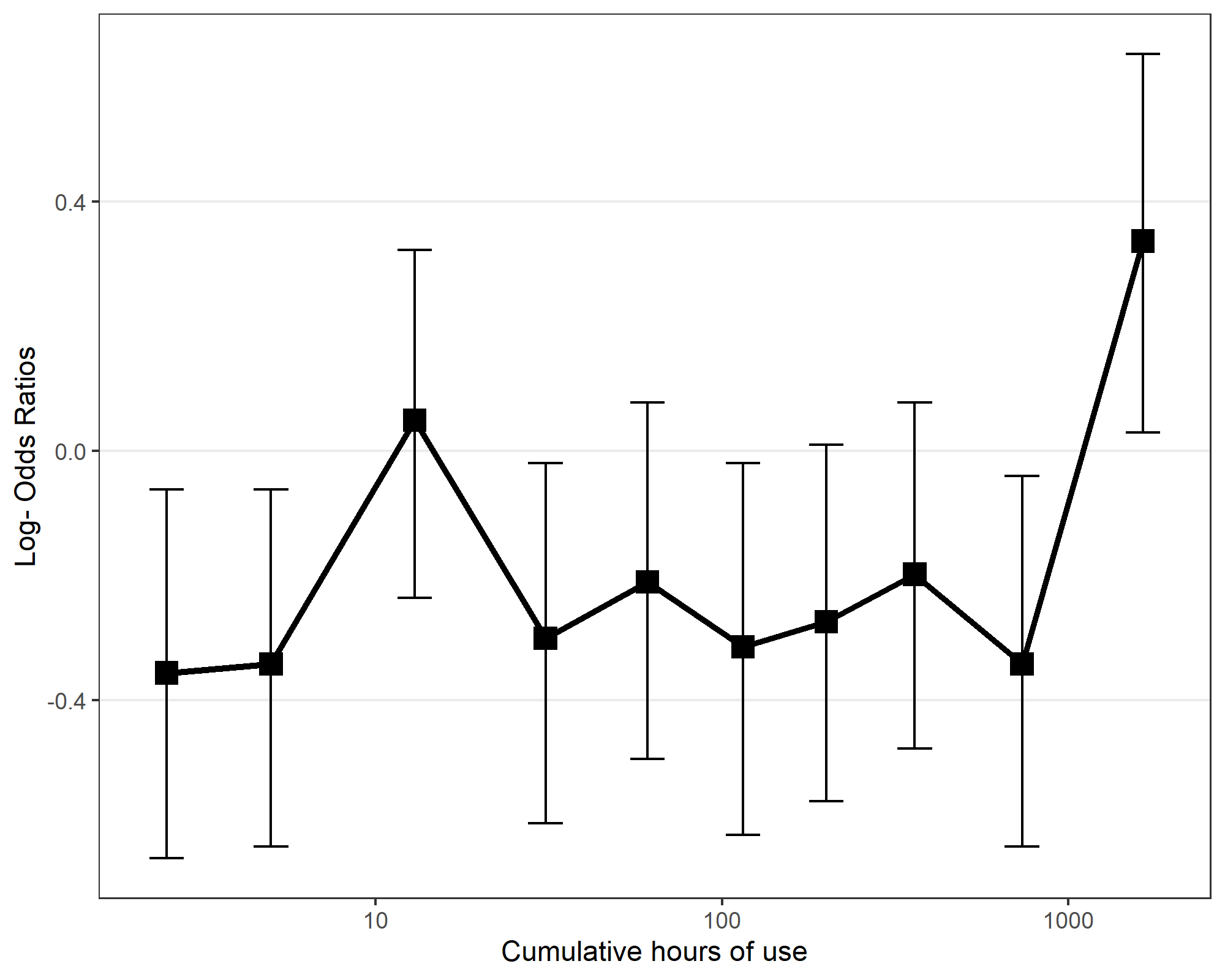

Supplement: Supplementary file 2 [file ede-35-437-s002.docx]
